# Prevalence of Hearing Loss After Open-Heart Surgery: A Systematic Review

**DOI:** 10.21470/1678-9741-2023-0197

**Published:** 2025-02-05

**Authors:** Khashayar Rezvani Emamzadehashemi, Ezzat Paryad, Arsalan Salari, Marzieh Jahani Sayad Noveiri

**Affiliations:** 1 Department of Neurosurgery, Faculty of Medicine, Imam Khomeini Hospital, Urmia University of Medical Sciences, Urmia, Iran; 2 Cardiovascular Diseases Research Center, Department of Cardiology, Heshmat Hospital, Department of Nursing, School of Nursing and Midwifery, Guilan University of Medical Sciences, Rasht, Iran; 3 Cardiovascular Diseases Research Center, Department of Cardiology, Heshmat Hospital, School of Medicine, Guilan University of Medical Sciences, Rasht, Iran

**Keywords:** Hearing Loss, Deafness, Cardiac Surgical Procedures, Systematic Review Study

## Abstract

**Introduction:**

Today, due to the increase in the incidence of heart problems in people, we see an increase in the number of heart surgeries worldwide. Hearing disorders in the form of hearing loss have been reported as one of the side effects of this kind of surgery in some studies. Considering the evidence of the effect of open-heart surgery on patients’ hearing loss, this systematic review study was conducted to determine the frequency of hearing loss after open-heart surgery.

**Methods:**

To carry out this systematic review, studies that investigated hearing loss after open-heart surgery in Iran and the rest of world, published in Persian or English, and various international electronic databases including PubMed®, Web of Science, Scopus®, and authentic Persian sources (*e.g.*, Irandoc and Scientific Information Database) until the end of 2021 have been indexed.

**Results:**

In the initial review, 46 of the 2230 initially searched articles had the conditions for further review. After checking the articles’ title and abstract and the working method of the studies and removing duplicates and incomplete articles, a total of 16 articles were included in the study. Then, their results were analyzed as the frequency of hearing loss after open-heart surgery by age and sex.

**Conclusion:**

The review of various studies confirms the fact that after open-heart surgery, the existence of evidence of hearing changes is not far from expected.

## INTRODUCTION

Hearing is one of the most important senses in humans, and any change in it will cause changes in health and quality of life. Six point eight million people in the world face hearing problems every year^[1]^. Normal hearing depends on the proper functioning of the conductive transmission system of the middle ear, the health of the biochemical and bioelectrical environment of the inner ear, and the proper functioning of the central nervous system and nuclei, and all of these depend on the normal functioning of the vascular, hematological, and endocrine systems and metabolic status, so any diseases affecting the mentioned systems can affect the hearing system of a person^[2]^. In general, hearing disorders are divided into two types: conductive hearing loss and sensorineural hearing loss (SNHL). SNHL is a set of common hearing disorders caused by dysfunction of the inner ear, auditory nerve, or auditory processing pathway in the central nervous system^[3]^. One of the complications after surgery is SNHL, which according to previous studies has a prevalence of one in every thousand people^[4,5,6,7]^, and often this hearing loss is sudden. The created hearing loss is not completely reversible and can cause problems for the patients^[8,9]^. This disorder is uncommonly observed after open-heart surgery when patients are placed under heart and lung bypass pumps^[5,10]^. Some sources believe that the cause of hearing disorders after admitting to the heart and lung bypass pump is caused by emboli originating from the cardiopulmonary pump device^[9,11]^. Therefore, the use of a heart and lung bypass pump, which is necessary to perform open-heart surgery, may be one of the vascular reasons for SNHL following the use of cardiopulmonary bypass, which increases the risk of sensorineural compromise^[11,12]^. As hearing loss after open- heart surgery is an uncommon complication, Walsted et al.^[13]^, in their study, mentioned only four cases of hearing loss, three right after the surgery and one a week later. Some researchers believe that the cause of hearing loss is the formation of an embolus or a decrease in inner ear blood supply^[13,14,15,16,17]^. In some studies, the incidence of severe hearing loss after open-heart surgery has been reported as 0.1%^[9,18]^. Also, studies have shown that factors such as the patient’s age, the decrease in central and peripheral body temperature while using the heart and lung pumps, as well as the drop in blood pressure while on these pumps, and the duration of pump usage can affect hearing after open-heart surgery^[4,11]^. Of course, some studies show that the role of open- heart surgery on hearing loss is weak. In his study, conducted on patients undergoing open-heart surgery in Turkey, Iriz concluded that performing coronary artery bypass does not bring the risk of hearing loss^[19]^. The lack of agreement in this field indicates the need for further investigations. Considering the increasing number of coronary artery bypass grafting in Iran and the world and the existence of evidence based on the effect of open-heart surgery on the hearing loss of patients, as well as studies based on the lack of effect of this surgery on the hearing status of patients, very limited research has been conducted in this field. Therefore, this review study was conducted to determine the frequency of hearing loss based on audiometric data after open-heart surgery.

## METHODS

### Approval, Data Sources, String, and Inclusion Criteria

The current research project has been approved by the Research Vice-Chancellor of Guilan University of Medical Sciences (IR.GUMS. REC.1401.191).

A systematic review was performed, in accordance with the Preferred Reporting Items for Systematic Reviews and Meta- Analyses (or PRISMA) guidelines.

The first step in this process was to search the following electronic databases: MEDLINE, PsycINFO®, CINAHL, ISI Web of Science, and Scopus®. Persian article databases (Scientific Information Database, Irandoc, and Iran Mag) were also examined. The string used was “Hearing Loss* OR Deafness AND Cardiopulmonary Bypasses”. No restrictions were used on the publication date or sample size. All articles that met the inclusion criteria were included. The second step was to examine and assess all the studies and reviews obtained on this topic, and finally, to perform the same operation for all the references cited in these studies. This stage was carried out in such a way that at first the title and summary of the article and the method of the study as well as the results were examined by two researchers, and the selection of articles was done with agreement.

The following inclusion criteria were applied: (a) published studies without a time limit until 2021; (b) studies that reported the percentage of hearing loss after open-heart surgery; (c) observational and interventional studies with hearing loss criteria based on audiometry; (d) the language used in the document (English, Persian, and studies from other countries that had English abstracts).

Studies in the form of case reports, letters to the editor, studies presented in congresses, qualitative studies, news, as well as studies whose results were not fully stated and articles that were not complete were excluded from the study. The studies found were listed and their compatibility with the criteria of this study was examined by two researchers separately.

### Coding of Results

The data collection form was designed and used for data extraction electronically. In case of not having access to the full text of the study, a request was made to receive the study from the central library of the university, and if it was not available, it was removed from the study. Two members of the team, independently, performed the search, selection, and detailed reading of the publications. In cases of disagreement, other researchers of the group were consulted as well.

The following variables were considered and recorded (Table 1): (a) authors; (b) year of publication; (c) country of publication; (d) language; (e) sample size; (f ) percentage of hearing loss after open-heart surgery; (g) sex and age of the sample population (mean, standard deviation, median or range). Methodological variables: (a) hearing loss measurement instrument; (b) design of the studies. Main outcomes of hearing loss after open-heart surgery: (a) results; (b) type of surgery; (c) time of connection to the pump and studies quality score.

**Table 1.** Summary of findings of included articles.

Author	Title/Design	Inclusion criteria	N/age/sex	Outcome measure	Results	Type of surgery	Time of connection to the pump	Quality score
1	Sanjay Kumar Munjal, Parul Malik, Anuradha Sharma, Naresh Kumar Panda, and Shyam K. Singh Thingnum (2013)^[17]^	Effects of Cardiopulmonary Bypass Surgery on Auditory Function: A Preliminary Study (Prospective- matched cohort)	Subjects with myocardial infarction scheduled to undergo CPB surgery.	30 Age range of 50-70 years; mean age was 40 years. 18 males (60.16%) and 12 females (62.23%).	Air and bone conduction thresholds were obtained 2 weeks after surgery for pure tone stimuli in a sound treated room at frequencies ranging from 250 to 4,000 Hz measured at each octave interval. Further, high frequency audiometry was performed up to 16 kHz. The lowest intensity, at which 50% of the spondee words were repeated correctly, was taken as speech reception threshold (SRT).	13 subjects had change in SNR of > 2 dB, and 6 had change of > 1 dB on TOAEs testing. On DPOAEs testing, a change of > 2 dB was observed in 11 subjects, and 8 subjects, had a change of 1dB. Hence, the findings revealed that 19 out of 30 subjects had changes in cochlear function.	Cardiac surgery with extracorporeal circulation	-	15
2	Mohsen Mirmohammad Sadeghi, Masoud Radman, Reza Bidaki, Mehdi Sonbolestan (2013)^[16]^	Sensorineural hearing loss in patients with coronary artery bypass surgery (Clinical trial)	All patients undergoing CABG in a 6-month period	105 87 males and 13 females. Mean age was 59.93 years.	Audiometric testing initially before the procedure to test the baseline hearing capacity; then 2 weeks after the procedure	The difference in threshold of hearing in frequencies of 250 Hz and 500 Hz was significant. Infrequency of 1,000 Hz in the right ear and in frequency of 4,000 Hz in the left ear was significant. We considered the level of bone conduction threshold of < 20 dB to be SNHL.	CABG	The mean time of connection to the pump was 85 minutes	18
3	Damghani M.A., Khodarahmi M., Shahidi A. (2008)^[2]^	Correlation between Coronary Artery Bypass Graft Surgery and Hearing Threshold Changes (Cross-sectional study)	All patients were candidates for CABG	65 47 males (72.3%) and 18 females (27.7%). Mean age in men was 56 ± 8.1 years and in women was 59.8 ± 6.2 years.	One week before surgery and 2 weeks after pure audio and impedance audiometry	Hearing threshold difference in the right ear at 4,000 Hz and in the left ear at 2,000 Hz was meaningful (P=0.027, P=0.004, respectively). Although hearing threshold differences at all frequencies and in both ears were greater in men than in women, the meaningful difference was only for the frequency of 1,000 Hz in the right ear (P=0.03) and 4,000 Hz in the left ear (P=0.034).	CABG	The mean time of connection to the pump was 92.9 ± 19.7 minutes	13
4	Erkan İriz, Metin Yılmaz, Bülent Gündüz, Ayşe İriz, Emrah Ereren, Yıldırım Ahmet Bayazit, Ali Yener (2009)^[19]^	Cardiopulmonary bypass circulation does not have adverse effects on ear functions: a study of otoacoustic emissions (Pre and postoperative assessments)	All patients were candidates for CABG without history of cerebrovascular disease such as ischemic cerebrovascular event, syncope, or HL	42 ears of 21 patients. 11 males and 10 females. Mean age was 61 years; age range 44-76 years.	Before and in the 6th day after CABG, PTA and speech discrimination tests were performed using an AC40 clinical audiometer and tympanometry and TEOAE and DPOAE tests	None of the patients had HL or sudden deafness after surgery. Preoperative and postoperative pure tone results of the patients did not differ significantly (*P*>0.05).	CABG	Cross-clamping time ranged from 36 to 71 minutes (mean 52 min.) and CPB time ranged from 55 to 155 minutes (mean 83 min.).	11
5	Khorsandi M T., Mohammadi M., Motasaddi Zarandy M., Mandegar M H., Yoosefnia M A., Sabetazad B. (2007)^[11]^	Audiometric changes after coronary artery bypass graft (Case series)	All patients were candidates for CABG	100 males. Age range 45-75 years.	Audiometric changes before and after surgery (hearing levels at multiple frequencies, SRT and speech discrimination score).	Those with slight changes ≤ 10 db (43 patients) and those having average deficits of > 10 db (10 patients).	CABG	The mean time of connection to the pump was 86 ± 5.04 minutes	11
6	Harvey M. Plasse, Frank C. Spencer, Myles Mittleman, and J. Ormond Frost (1980)^[18]^	Unilateral sudden loss of hearing An unusual complication of cardiac operation (Cohort study)	All patients with cardiac surgery	7,000. The 7 patients with this problem were all men. Age range 25-70 years.	HL was confirmed by audiogram and was found to be sensorineural.	In 7 of these patients, sudden loss of hearing in one ear developed immediately after the operation. Four of the 7 patients showed improvement in hearing after the initial loss, although in no case did the hearing return completely to normal.	Tricuspid valve replacement; repair of atrial septal defect/double coronary artery bypass/quadruple coronary artery bypass	Bypass time mean 2 hours and 42 min. Aortic cross- clamping time mean 1 hour and 15 min.	7
7	M. Casale, M. Potena, V. Rinaldi, M. Lusini, E. Vesperini, M. Chello, E. Covino, F. Salvinelli (2011)^[20]^	Evaluation of ear function after cardiopulmonary bypass with otoacoustic emissions: a pilot study (Pre and postoperative assessments)	All patients who underwent open-heart surgery	10. 5 males and 5 females.	Clinical otological examination, a pure tone audiogram, impedance audiometry, and otoacoustic emissions on the day before surgery (preoperative phase) and 72 hours after surgery (postoperative phase).	No significant differences were found between pre and postoperative audiological assessment both in hearing level and in otoacoustic emissions.	Coronary bypass/aortic valve substitution/mitral valve substitution	Cross-clamping time mean 54.4 min. CPB time ranged from 65 to 93 min. (mean = 82 min.)	11
8	A Iriz, K Cagli, C Gocer, E Dursun, H Korkmaz, A Eryilmaz (2008)^[21]^	"Effects of open- heart surgery on hearing thresholds measured by high frequency audiometry (Pre and postoperative assessments)"	All patients undergoing open-heart surgery	20. 5 females and 15 males.	Audiometric measurements were compared both pre and postoperatively.	Patients' pre and postoperative pure tone audiometric results were significantly different at some frequencies (*P*<0.05). In addition, there was a significant impact of hypertension, hypercholesterolemia, history of myocardial infarction, and cross- clamping time.	Coronary artery bypass surgery or valve surgery	The mean duration of total cardiac bypass time was 66.45 minutes (SD 23.3), the mean cross-clamping time was 40.5 minutes (SD 16.8)	13
9	Barlas N. Aytacoglu, Cengiz Ozcan, Nehir Sucu, Kemal Gorur, Oben Doven, Handan Camdeviren, Necmi Köse, Murat Dikmengil (2006)^[12]^	Hearing loss in patients undergoing coronary artery bypass grafting with or without extracorporeal circulation (Before after assessment)	All patients who underwent CABG with or without extra corporeal bypass pump (20 with extracorporeal bypass pump and 17 without it.)	37. 20 males and 17 females.	The mean hearing thresholds were determined with PTA at six frequencies (250, 500, 1,000, 2,000, 4,000, and 8,000 Hz), and speech audiometry (speech discrimination scores, speech recognition thresholds) in all patients, in a soundproof room, before and in the 3rd day after surgery.	Hearing threshold changes were detected in 9 group I patients (45%) and 3 group II patients (17.65%). The difference between the two groups was statistically significant (*P*=0.0426). Most of the changes, as assessed in the audiograms, were found to be between the frequencies of 4,000 and 8,000 Hz. HL was found to be bilateral in 5 of the 9 patients in group I (55.5%) and in 1 of the 3 patients in group II (33.3%).	With extracorporeal circulation (20) Without extracorporeal circulation (17)	Total extracorporeal circulation time 123.05 ± 43.33 minutes Cross-clamping time 64.80 ± 32.09 minutes	13
10	Richmond Jay Brownson, Malcolm H. Stroud, and William F. Carver (1971)^[22]^	Extracorporeal Cardiopulmonary Bypass and Hearing (Prospective study)	All patients who underwent open-heart surgery	50	Pure-tone tests and a speech audiometer for speech tests or a model for both pure-tone and speech tests.	In the 50 patients examined both preoperatively and postoperatively, no significant change in hearing was documented which could be attributed to micro embolism.	Extracorporeal CPB	The length of time on bypass varied from 21 to 250 minutes, the average being 102 minutes.	11
11	Donne, A. J., Waterman, P., Crawford, L., Balaji, H.P., Nigam, A. (2006)^[14]^	A single-blinded case controlled study on effects of cardiopulmonary circulation on hearing during coronary artery bypass grafting (Single-blinded case controlled study)	All patients undergoing open-heart surgery.	52	PTA performed on 0–1 day preoperatively and 5–7 days postoperatively	No difference between the area generated between mean pre and postoperative audiograms (*P* = 0.754). No significant difference between off- *vs.* on-pump CABG was demonstrated for average differences at 250–500 Hz, 4 kHz, 4–8 kHz, and 8 kHz, and no difference between right and left ears for each individual frequency.	14 control patients undergoing off-pump CABG and 38 study patients undergoing on-pump CABG	-	20
12	J. J. Phillipps and A. R. D. Thornton (1996)^[4]^	Audiometric changes in patients undergoing coronary artery bypass surgery (Case control)	All patients who underwent CABG with (case group) and without (control group) CPB, all without medical history of hearing problem.	20	Audiometry on day before CABG and in the 6th day after CABG	From the bypass sample of 40 ears, 5 ears (4 individuals) had an individually statistically significant HFHL.	CPB	Mean duration operation 73 min.	11
13	Lalitha Gopineti et al. (2019)^[23]^	Prevalence of Sensorineural Hearing Loss in Children with Palliated or Repaired Congenital Heart Disease (Retrospective study)	Children who underwent congenital heart surgery.	172. 79 males (63.2%) (15 with HL [75%]). 46 females (36.8%) (5 with HL [25%]).	HL was classified as mild when the HL range was 26-40 dB. HL range in > 40 dB was grouped as moderate to severe to profound loss.	A total of 172 patients were identified, 20 of whom were found to have hearing impairment ranging from mild loss to moderate to severe to profound loss in one or both ears.	Surgeries related to congenital heart disease	-	12
14	Madison A. Grasty., et al. (2018)^[24]^	Hearing Loss after Cardiac Surgery in Infancy: an Unintended Consequence of Life-saving Care (Prospective observational study)	Children with ≤ 6 months of age, undergoing CPB, with or without deep hypothermic circulatory arrest who completed a standard audiologic evaluation as part of a comprehensive neurodevelopmental evaluation at 4 years of age.	348. 165 females (43.3%) (32 with HL [42.7%]). 216 males (56.7%) (43 with HL [57.3%]).	Thresholds were obtained for pure tone air conduction stimuli at frequencies of 250-8,000 Hz in an audiometric booth. Normal hearing sensitivity was defined as response thresholds of ≤ 15 dB HL. Pure tone average: 21-39 dB HL (mild); 40-54 dB HL (moderate); 55-69 dB H (moderate to severe); 70-89 dB HL (severe); and ≥ 90 dB H (profound). HFHL was defined as confined to the region at ≥ 2,000 Hz.	75 of the 348 children enrolled in the study were diagnosed with HL, resulting in a prevalence estimate (95% CI) of 21.6% (17.2, 25.9). The prevalence rates of conductive HL, SNHL, and indeterminate HL were 12.4% (8.8, 16.0), 6.9% (4.1, 9.7), and 2.3% (0.6, 4.0), respectively. Of the 75 children with HL, 50 (67.6%) had mild HL. Of the 24 with SNHL, 12 (50%) had moderate to severe HL and 8 (33.3%) had HFHL. Only 18 (5.23%) parents had reported a diagnosis of HL in their infants prior to this evaluation, 10 of whom used hearing technology.	Bypass	Total bypass time at 1st operation 65.7 min. (39.3)	13
15	Taghadomi . et al. (2011)^[25]^	Effect of cardiopulmonary pump on hearing loss after coronary artery bypass surgery (Case control study)	All patients undergoing open-heart surgery.	200. 124 males (62%) and 76 females (38%). Mean age was 57 ± 10 years.	Otoacoustic emission test was performed on the patients who underwent CPB surgery the day before the operation, the day after the operation, and in case of HL after a week.	The rate of HL after bypass surgery was 12%. The rate of final HL was 14.3% in the group operated with a pump and 6.7% in the group without a pump (*P*=0.09). In the examination of the relationship between the preoperative hearing test and the final hearing test in the pump operation group, there was a significant relationship (*P*<0.005). The effect of the pump during heart surgery on HL after surgery was here loss of 18.6% in men in the operation group with pump (*P*=0.002), hear loss of 7.4% in women in the operation group with pump, and hear loss of 18.2% in the operation group without pump (*P*=0.1). In patients with HL, it was 4.2% at 1,500 and 2,000 Hz, 12.5% at 2,500 and 3,000 Hz, and 83.3% at 3,500 and 4,000 Hz.	CPB	The average length of surgery in this study was 289 ± 82 minutes	13
16	Karin T. Bork et al. (2018)^[26]^	Prevalence of Childhood Permanent Hearing Loss after Early Complex Cardiac Surgery (Prospective observational study)	All heart surgeries	691 children. 445 males (64.4%); 26 HL (63.4%).	Audiology follow-up by registered pediatric-experienced audiologists at 6-8 months after surgery, age of 2 years, and as required throughout and thereafter to complete diagnoses. PHL at any frequency (500-4,000 Hz) is defined as responses of > 25-decibel hearing level in either ear. PHL was evaluated by type (conductive or sensorineural), pattern (flat or sloping), and severity (mild to profound).	41 children had PHL (5.9%) (95% CI 4.3%, 8.0%). By cardiac defect, prevalence was biventricular, 4.0% (95% CI 2.5%, 6.1%); single ventricle, 10.8% (95% CI 6.8%, 16.1%). 87 (12.6%) of 691 had syndromes/genetic abnormalities with known association with PHL; of these, 17 (41.5%) had PHL. Of 41 children, 4 had permanent conductive, moderate to severe loss (1 bilateral); 37 had moderate to profound sensorineural loss (29 bilateral with 20 sloping and 9 flat), 6 with cochlear implant done or recommended.	CPB	CPB time: 113.4 (47.3) min	12

CABG=coronary artery bypass grafting; CI=confidence interval; CPB=cardiopulmonary bypass; DPOAE=distortion product otoacoustic emissions; HFHL=high-frequency hearing loss; HL=hearing loss; PHL=pediatric hearing loss; PTA=pure tone audiometry; SD=standard deviation; SNHL=sensorineural hearing loss; SNR=signal to noise ratio; SRT=speech reception threshold; TOAE=transient evoked otoacoustic emission

### Quality Appraisal

A descriptive analysis was made of the study variables thus included, concerning the quality of each publication selected; to check the quality of observational articles, a modified Strengthening the Reporting of Observational Studies in Epidemiology (STROBE) tool was used. For interventional studies, a modified Consolidated Standards of Reporting Trials (CONSORT) tool was used.

## RESULTS

**Fig. 1:**
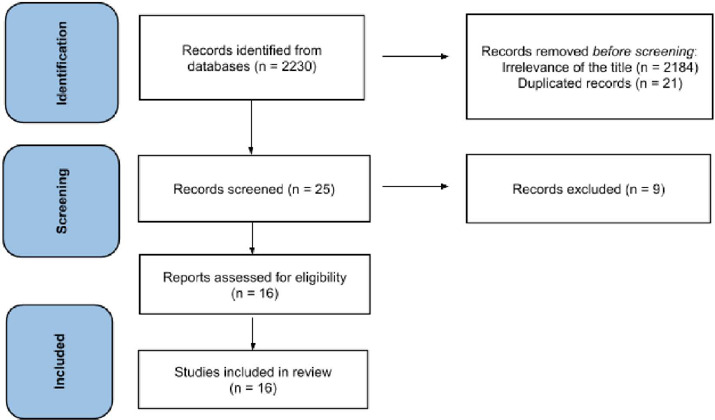
Flowchart of articles screening process

During the initial search, 2230 articles were found with the investigated keywords. After reviewing the titles of the studies and their abstracts, 46 articles entered the next stage of review. Of these, 21 articles were removed due to duplication. The text of 25 articles was read completely, and five articles were excluded due to lack of inclusion criteria (study design or lack of access to its full text, which included a thesis and three article abstracts). Finally, 16 articles were selected for data analysis. The characteristics of these studies are presented in Table 1. Out of 16 articles, three were related to children undergoing open-heart surgery, which included 1211 children aged between two and five years. There were 13 articles related to adults, which included 7712 patients with an age range of 20 to 81 years in terms of hearing loss after open-heart surgery (Figure 1). In nine articles, the samples were analyzed by sex. In these nine studies, 434 patients were male, and 156 patients were female. In a total of 8923 patients undergoing open-heart surgery, hearing loss was reported in 431 (4.8%) patients.

The minimum duration of surgeries reported in these studies was 21 minutes, and the maximum was 289 minutes.

## DISCUSSION

In order to review the results of studies that investigated the changes in hearing levels after open-heart surgery and answer the question of whether open-heart surgery can affect the hearing of patients, 7712 patients in the age range 20-81 years and 1211 patients in the age range 2-5 years were included in this systematic review study. In 12 out of 16 articles, the findings indicated a significant difference in hearing before and after open- heart surgery. In general, in a total of 8923 children and adults undergoing open-heart surgery, hearing loss was reported in about 5% of these patients. Considering this issue, it seems that hearing loss after open-heart surgery can be a worrying issue. Therefore, special attention should be paid to hearing status and its possible changes as one of the complications of open-heart surgery. The results of some studies showed that increasing the duration of the patient’s stay on the cardiopulmonary bypass pump can affect the occurrence of hearing loss in these patients^[2,4]^. Some studies have also pointed to factors such as the duration of aortic cross-clamping, the presence of some underlying diseases, simultaneous coronary artery transplant surgery, and heart structural surgery in the occurrence of more hearing changes following heart surgery^[17,20,21]^.

Also, the frequency of hearing loss in children aged two to five years after open-heart surgery is estimated to be about 21.6% in this study, which is very high in a population with a high life expectancy. Special attention and follow-up of this complication in this group are required. Human hearing is in the frequency range of 20 to 20,000 Hz, and in most cases, hearing loss after open- heart surgery affects the maximum hearing range, thus reducing the patient’s ability to hear sounds with frequencies higher than 1,000 Hz^[2]^. In the case of patients who have been under the heart bypass pump for a longer period of time, a greater decrease in hearing range has been observed^[2,22]^. During the period when patients undergo cardiac bypass for open-heart surgery, using a heart and lung pump bypass, the blood pressure is maintained at about 60 mmHg or even less^[23]^. Despite the decrease in body temperature and in the metabolism affected by it, the decrease in blood pressure may be associated with the decrease in blood supply to some areas of the central nervous system, and the change in senses after heart surgery is affected by the decrease in blood supply to these areas. Since the inner ear needs an oxygen-rich blood supply to function normally, insufficient blood flow in the inner ear can contribute to hearing loss. The function of auditory cilia in the cochlea requires proper blood circulation, and the decrease in oxygen supply following the decrease in blood pressure may affect their function. These conditions may be associated with hearing loss after open-heart surgery^[24,25,26,27,28]^. For this reason, using a cardiopulmonary bypass pump for a long period of time during open-heart surgery can provide the conditions for the hearing loss of patients. Hearing loss can be classified as SNHL, conductive hearing loss, or a combination of the two. SNHL is more common than other types and occurs when the auditory nerve or the cilia inside the cochlea do not receive adequate blood supply^[25,29]^. In a study written about the effect of open-heart surgery on patients' hearing, it is emphasized that the decrease in sensorineural hearing in patients undergoing open-heart surgery is not complete or severe^[11]^.

Although the change in hearing level after open-heart surgery does not happen in all patients, it should be considered even in a small percentage of patients because hearing is one of the main senses, and the change in this ability can cause patients to face many problems after surgery. Due to the lack of available studies on this condition, it seems that the information about it is also insufficient. Perhaps it is for the same reason that the necessary assessments on the comparison of hearing before and after surgery are not carried out in almost any cardiac surgery center. Another important finding in the present study is the difference in the change of hearing ability according to sex; in most studies, the change of hearing threshold in all frequencies and both ears is more usual in men than in women. The results of the study by Wang et al.^[31]^ also showed that after men and women were exposed to noise, the hearing loss of men was four times more than that of women. Some studies have suggested a possible reason for this difference in the protective role of estrogen in women^[2,32,33]^.

The findings of this study have confirmed hearing changes in some patients undergoing open-heart surgery. Based on this, it can be suggested that one of the preoperative cares before open- heart surgery is to check the hearing level of patients. In addition, many patients undergoing coronary artery transplant surgery suffer from diabetes, which can be associated with the destruction of the end vessels that supply blood to the central and peripheral nerves.

### Limitations

Because the number of studies conducted on hearing changes following open-heart surgery is minimal, and underlying diseases such as diabetes, which seems to affect the results of surgeries, are not mentioned in these studies, it was not possible to investigate the effect and draw conclusions about it in the present study, which can be a limitation. In addition, due to the dispersion of the methods used in the studies included in the current research and their scattered results, meta-analysis was not possible, which could be another limitation of the current study.

## CONCLUSION

The review of various studies confirms the fact that after open- heart surgery, the existence of evidence of hearing changes in patients is not far from expected, and based on this, it can be suggested that one of the cares in patients who are candidates for open-heart surgery is to check their hearing levels before the operation.

## Abbreviations, Acronyms & Symbols

CABG: Coronary artery bypass grafting
CI: Confidence interval
CPB: Cardiopulmonary bypass
DPOAE: Distortion product otoacoustic emissions
HFHL: High-frequency hearing loss
HL: Hearing loss
PHL: Pediatric hearing loss
PTA: Pure tone audiometry
SD: Standard deviation
SNHL: Sensorineural hearing loss
SNR: Signal to noise ratio
SRT: Speech reception threshold
TOAE: Transient evoked otoacoustic emission
